# Assessment of Population Well-being With the Mental Health Quotient: Validation Study

**DOI:** 10.2196/34105

**Published:** 2022-04-20

**Authors:** Jennifer Jane Newson, Vladyslav Pastukh, Tara C Thiagarajan

**Affiliations:** 1 Sapien Labs Arlington, VA United States

**Keywords:** psychiatry, public health, methods, mental health, population health, social determinants of health, global health, behavioral symptoms, diagnosis, symptom assessment, psychopathology, mental disorders, mHealth, depression, anxiety, attention deficit disorder with hyperactivity, autistic disorder, internet

## Abstract

**Background:**

The Mental Health Quotient (MHQ) is an anonymous web-based assessment of mental health and well-being that comprehensively covers symptoms across 10 major psychiatric disorders, as well as positive elements of mental function. It uses a novel life impact scale and provides a score to the individual that places them on a spectrum from Distressed to Thriving along with a personal report that offers self-care recommendations. Since April 2020, the MHQ has been freely deployed as part of the Mental Health Million Project.

**Objective:**

This paper demonstrates the reliability and validity of the MHQ, including the construct validity of the life impact scale, sample and test-retest reliability of the assessment, and criterion validation of the MHQ with respect to clinical burden and productivity loss.

**Methods:**

Data were taken from the Mental Health Million open-access database (N=179,238) and included responses from English-speaking adults (aged≥18 years) from the United States, Canada, the United Kingdom, Ireland, Australia, New Zealand, South Africa, Singapore, India, and Nigeria collected during 2021. To assess sample reliability, random demographically matched samples (each 11,033/179,238, 6.16%) were compared within the same 6-month period. Test-retest reliability was determined using the subset of individuals who had taken the assessment twice ≥3 days apart (1907/179,238, 1.06%). To assess the construct validity of the life impact scale, additional questions were asked about the frequency and severity of an example symptom (*feelings of sadness, distress, or hopelessness*; 4247/179,238, 2.37%). To assess criterion validity, elements rated as having a highly negative life impact by a respondent (equivalent to experiencing the symptom ≥5 days a week) were mapped to clinical diagnostic criteria to calculate the clinical burden (174,618/179,238, 97.42%). In addition, MHQ scores were compared with the number of workdays missed or with reduced productivity in the past month (7625/179,238, 4.25%).

**Results:**

Distinct samples collected during the same period had indistinguishable MHQ distributions and MHQ scores were correlated with *r*=0.84 between retakes within an 8- to 120-day period. Life impact ratings were correlated with frequency and severity of symptoms, with a clear linear relationship (*R*^2^>0.99). Furthermore, the aggregate MHQ scores were systematically related to both clinical burden and productivity. At one end of the scale, 89.08% (8986/10,087) of those in the Distressed category mapped to one or more disorders and had an average productivity loss of 15.2 (SD 11.2; SEM [standard error of measurement] 0.5) days per month. In contrast, at the other end of the scale, 0% (1/24,365) of those in the Thriving category mapped to any of the 10 disorders and had an average productivity loss of 1.3 (SD 3.6; SEM 0.1) days per month.

**Conclusions:**

The MHQ is a valid and reliable assessment of mental health and well-being when delivered anonymously on the web.

## Introduction

### Background

The World Health Organization defines mental health as “a state of well-being in which the individual realizes his or her own abilities, can cope with the normal stresses of life, can work productively and fruitfully, and is able to make a contribution to his or her community” [1]. On the basis of this definition, assessments of mental health should reflect the presence of dysfunction and also provide insight into the positive aspects of mental functioning [2-5]. However, the clinical heritage of mental health assessment means that most assessment tools are built around specific psychiatric disorder categories taken from the clinical classification systems of the Diagnostic and Statistical Manual of Mental Disorders, Fifth Edition (DSM-5) [6], or the International Classification of Diseases [7] and, therefore, are not designed to provide a perspective on the continuum of mental health and well-being across the general population. In contrast, assessments of mental health that are more relevant across the spectrum of the general population can support the early identification of at-risk individuals before symptoms escalate, improve uptake in help-seeking behaviors, and reveal relevant social determinants to support the active management of mental health and well-being through self-care behaviors and preventative strategies and interventions at various scales, from organizations to countries [8-10].

Population-level assessments also provide an opportunity for understanding the scale of mental health challenges at a global level. For example, in 2017, 792 million people were estimated to be living with a mental health disorder worldwide [11], whereas depression is the leading cause of disability as measured by years lived with disability [12]. In addition, suicide was the fourth leading cause of death among people aged 15-29 years worldwide in 2019 [13] and is still poorly understood [14,15]. However, presently, there are few reliable and valid tools that can provide an aggregate and measurable view across the full spectrum of a global population from distressed to thriving as well as estimating clinical burden in an aggregate, disorder-agnostic way. Furthermore, as mental health and well-being can change substantially based on external circumstances, as evidenced by the COVID-19 pandemic [16-18], it is important to have metrics that track the extent and nature of these changes and their impact on clinical burden as well as on the productive capacity of a population. For example, evidence suggests a clear relationship between mental health and well-being and productivity, resulting in both absenteeism and presenteeism [19-25], an increased prevalence of burnout [26,27], and significant personal and economic loss [28-32]. This further highlights the importance of preventative measures for actively managing the mental health and well-being of working-age adults in the general population.

To address this need for a population-based, disorder-agnostic assessment that spans the spectrum of mental health and well-being and for a single measurable metric of mental health and well-being, we developed a new web-based assessment tool delivering a metric called the Mental Health Quotient (MHQ) [33]. The MHQ assesses the complete breadth of mental health elements spanning the range from symptoms to positive mental assets using a unique life impact scale and aims to enable a paradigm for managing and improving the lives and well-being of all people, not just those with a clinical disorder.

### The MHQ Assessment

The MHQ was developed based on a comprehensive review of symptoms by coding questions across 126 commonly used psychiatric assessment tools spanning depression, anxiety, bipolar disorder, attention-deficit/hyperactivity disorder, posttraumatic stress disorder, obsessive-compulsive disorder (OCD), addiction, schizophrenia, eating disorders, and autism spectrum disorder as well as cross-disorder tools (see Newson et al [34] for a complete list of assessment tools). These disorders were selected based on a review of the disorders included in the Structured Clinical Interview for the DSM-5, Clinician Version [35]. In addition, autism spectrum disorder and eating disorders were included because of both their prevalence and their broad public and scientific interest. Symptoms from these 126 assessments were consolidated into a set of 43 symptom categories and reviewed and expanded in the context of the Research Domain Criteria constructs put forward by the National Institute of Mental Health [36-38]. The resultant 47 elements were then split into two formats: mental functions that could manifest as a spectrum from positive to negative (spectrum questions) and those symptoms that purely represented detractions from overall mental health (problem questions).

Spectrum and problem questions within the MHQ are answered using a 9-point scale reflecting the consequences on one’s life functioning and impact on their ability to carry out tasks and activities in their daily life. Therefore, the scale is different from traditional mental health assessments, which typically focus on the frequency, severity, duration, or timing of symptoms [34]. An aggregate MHQ score developed using an algorithm that nonlinearly transforms the life impact scale based on different categories of symptom seriousness is provided on completion of the assessment [33]. This score is intended as a representation of the overall mental health and well-being of the individual and is categorized from Distressed (−100) to Thriving (+200).

The MHQ is currently used on the web as part of an open data project called the Mental Health Million Project, which is a web-based platform that monitors the status of population mental health across the globe and currently spans 30 countries and 4 languages (English, French, Spanish, and Arabic). In this paper, we evaluate the potential of the MHQ to be used as a valid and reliable measure of mental health and well-being both at the individual and population levels to determine how mental health and well-being evolves over time across the globe and the impact of these changes on clinical burden and productivity. We aim to address (1) how the unique MHQ life impact scale relates to more commonly used metrics of frequency and severity; (2) whether an anonymous web-based assessment serves as a true measure of the population by demonstrating the population and test-retest reliability of the MHQ; and (3) how well the composite MHQ score relates to functional criteria such as clinical diagnostic criteria, workdays missed, and overall life productivity. We hypothesize that the MHQ will show good validity and reliability as an assessment of mental health and well-being when delivered anonymously on the web.

## Methods

### Recruitment of Participants

The data were taken from the Mental Health Million open-access database [39] and included responses from 179,238 English-speaking individuals from the United States, Canada, the United Kingdom, Ireland, Australia, New Zealand, South Africa, Singapore, India, and Nigeria collected during 2021. Participants were recruited via outreach campaigns on Facebook and using Google Ads by targeting a broad cross-section of adults aged 18-85 years across a wide geographic and socioeconomic demographic. The anonymous assessment was freely available on the web for anyone to complete, and individuals took the assessment for the purpose of obtaining their personalized mental health and well-being report on completion. The provision of a personal report aimed to ensure greater interest of the respondent in answering questions thoughtfully and accurately. Only respondents who found the assessment easy to understand (ie, responded *Yes* to the question *Did you find this assessment easy to understand?*) were included in the analysis. This resulted in the exclusion of 2.58% (4620/179,238) of respondents, leaving 174,618 for the full analysis.

### Ethics Approval

The study received ethics approval from the Health Media Lab Institutional Review Board (Office for Human Research Protections Institutional Review Board #00001211, Federal Wide Assurance #00001102, IORG #0000850).

### Assessment of Reliability

#### Reliability Across Randomly Selected Samples

All respondents from the United States, India, Australia, and the United Kingdom between January 2021 and June 2021 were pooled together (44,132/174,618, 25.27%). A total of 4 randomly selected and nonoverlapping samples of 11,033 people with similar demographic composition were selected. The average rating (1-9) of each MHQ-scored element for each sample, the average MHQ score for each sample, and the statistical differences between the samples were then computed.

#### Internal Consistency Analysis

The MHQ is designed to be as parsimonious as possible without repetition. However, the internal consistency of the MHQ was evaluated by looking at the relative correlations between elements that would be expected to be correlated compared with those that would not. First, the correlation between 2 questions about sleep quality within the MHQ was computed (N=174,618). Sleep question 1 asked respondents to *Assess your sleep quality*, and sleep question 2 asked respondents: *In general, I get as much sleep as I need*. The 1-9 rating score from sleep question 1 was correlated with the transformed answers to sleep question 2, where each answer option was assigned a number that was roughly equivalent to the text description: *all the time*=7, *most of the time*=5, *some of the time*=3, and *hardly ever*=1. Second, the correlation between 2 questions about mood was computed. Mood question 1 asked respondents to *Assess your feelings of sadness, distress, or hopelessness* and was rated on a 1-9 life impact scale. Mood question 2 asked respondents *How would you describe your overall mood right now?* and was rated on a 1-9 scale from *Very negative* to *Very positive*. Comparisons were also made between responses to the related MHQ elements of *Self-worth and confidence* and *Self-image*. In addition, comparisons were made between the elements of *Physical intimacy* and *Memory*, *Emotional control* and *Coordination*, and *Memory* and *Emotional control*, which would not be expected to have a significant correlation.

#### Test-Retest Reliability

The MHQ is designed to measure changes in the mental health and well-being of the population and, therefore, in individual mental health and well-being status over time. Therefore, the MHQ scores of individuals could change over time. However, over short time frames of less than a year, most individuals would not be expected to change significantly. Within the sample of 174,618 respondents, email addresses were provided by 80,955 (46.36%) to receive their MHQ report. These email addresses were automatically converted into anonymous unique identifiers to identify repeat respondents. Of these 80,955 respondents, 2231 (2.76%) had taken the MHQ twice at varying time intervals up to 15 months from the time of the first assessment. Those who took the MHQ twice within the same day or immediately the next day were excluded as they were more likely to be experimenting with answer choices than evaluating their own change over time in an honest way. Thus, only those who had at least 3 days between attempts were included in the analysis (1907/2231, 85.48%). We examined the test-retest reliability of the MHQ in this sample by looking at the correlation between the element ratings on the first and second attempts as well as the correlation between MHQ scores across both attempts.

### Validation of the Life Impact Scale

Clinical assessments are heterogeneous in their evaluation of the frequency and severity of symptoms. For example, a review of 126 assessment tools found that, across 19 commonly used depression scales, 51% of questions asked about frequency of symptoms and 32% asked about severity, whereas, across 9 posttraumatic stress disorder assessment tools, 17% of questions asked about frequency and 53% asked about severity [34]. Given the lack of a clear understanding of which aspect (eg, frequency or duration) of a symptom matters most, the MHQ uses a 9-point life impact scale reflecting the impact of a particular mental aspect on one’s ability to function [33]. For example, for questions pertaining to mental health challenges, 1 referred to *Never causes me any problems*, 9 referred to *Has a constant and severe impact on my ability to function*, and 5 referred to *Sometimes causes me difficulties or distress but I can manage*. For the purpose of validation, for the question that asked individuals to rate the impact of their *Feelings of sadness, distress, or hopelessness* on this 9-point scale, two additional questions were asked when a value of ≥5 was selected: (1) *How many days in the last week did you experience these feelings?* with options for selection from 0-7 (similar to the format in depression screening tools such as the Center for Epidemiologic Studies–Depression scale [40,41]) and (2) *On these days, how did these feelings impact your ability to function in life?* with five options of increasing severity—1=*They would come and go while I went about my life as normal*; 2=*I did what I had to do, but they were always there in the back of my mind*; 3=*I managed but it took extreme effort*; 4=*They stopped me doing the things I usually do, or would want to do*; and 5=*They consumed me so much I was unable to get out of bed*. The average frequency and severity and the standard error of measurement (SEM) were then computed for each selection from 5 to 9 on the life impact scale.

### Relationship Between MHQ Score and Clinical Burden

The computation of the MHQ score takes into account the number of severe symptoms (ie, those scored as having a highly negative life impact). Thus, the number of elements with a rating that signifies a highly negative life impact decreases as the MHQ score increases, although the nonlinear weighting differs for different types of symptoms [33]. To assess how effectively the MHQ score relates to clinical burden, we mapped elements of the MHQ to the diagnostic criteria for each of the 10 major DSM-5 disorders on which the MHQ is based (see *The MHQ Assessment* section) and examined (1) the percentage of individuals meeting the diagnostic criteria for at least one disorder and (2) the average number of diagnoses per person for each MHQ score bin of 25. Full details of the thresholds used to determine the presence of a clinical symptom and the mapping to the DSM-5 disorder criteria are described in Newson et al [42]. In brief, MHQ elements were first mapped to the symptoms described within the diagnostic criteria for each of the 10 DSM-5 disorders based on the closest semantic match. For each of the 47 MHQ elements, responses were determined to be clinically significant symptoms if they met a particular threshold of impact on the individual’s ability to function, approximately equivalent to experiencing the symptom 5 days a week (≥8 for problem elements and ≤1 for spectrum elements). The specific diagnostic criteria rules of the DSM-5 (eg, must be experiencing ≥5 symptoms) were then applied to arrive at a disorder diagnostic mapping. A set of rules using combinations of the DSM-5–mapped MHQ elements was then developed to align with these criteria descriptions for each of the 10 disorders. For each respondent (N=174,618), these rules were applied to their MHQ clinical symptom profile to determine the diagnostic match to each of the 10 disorders. See Newson et al [42] and the *Limitations* section below for further discussion of this approach.

### Relationship Between MHQ Score and Productivity Criterion

To assess the relationship between the MHQ score and measures of functional productivity, a subset of participants (7625/174,618, 4.37%) were asked two additional questions: (1) *How many days during the past month were you totally unable to work or carry out your normal activities because of problems with your physical or mental health* and (2) *How many days during the past month were you able to work and carry out your normal activities, but could not get as much done because of problems with your physical or mental health?* with options to select a number between 0 and 31. Individuals were then grouped by MHQ score in bins of 25, and the average and SEM of days of work missed (*M*) and days with reduced productivity (*R*) were then computed for each bin. We then computed the overall loss of life productivity for each individual as *M* + *n* *R, where *n* represented an assumed loss of productivity on those days ranging from 20% to 50%. Data were examined for all respondents together and for a subset of respondents who answered *Employed/Self-Employed* to the MHQ question *Please select which best describes your occupational status?* (alternative answer options included *Homemaker*, *Unemployed*, *Retired*, *Studying*, and *Not able to work*).

## Results

### Reliability and Internal Consistency in the MHQ

#### Assessment Reliability

**Figure 1:**
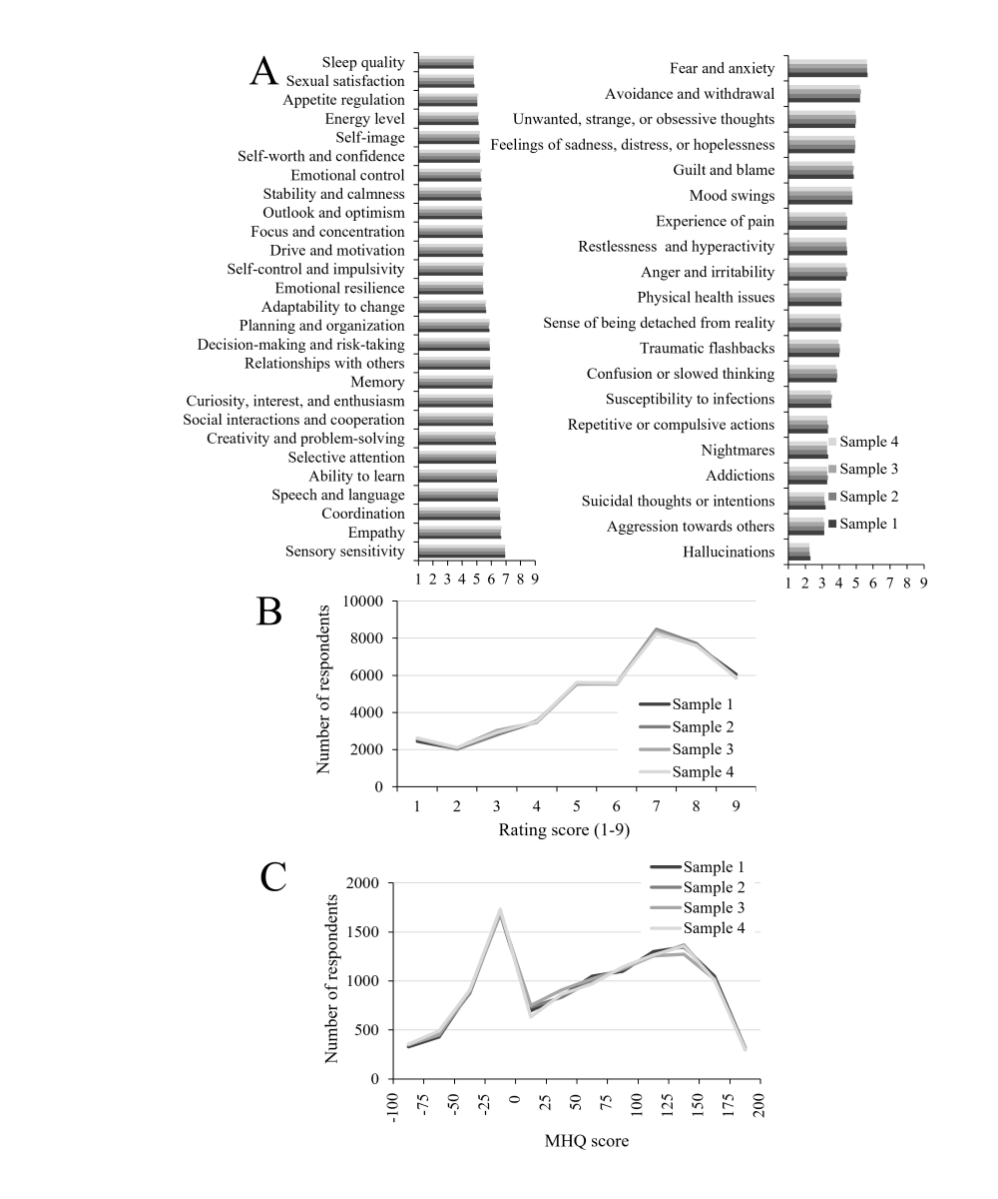
Reliability of the Mental Health Quotient (MHQ). (A) The average ratings of each of the 27 MHQ spectrum elements (left) and 20 MHQ problem elements (right) in 4 separate samples of the MHQ obtained over a similar period were indistinguishable (each bar is a sample). (B) Distribution of ratings for an example MHQ element (Self-image) in each of the 4 samples (each line is a sample). (C) Distribution of MHQ scores in each of the 4 samples (each line is a sample) for an example MHQ element (Self-image).

Figure 1A shows the average rating for each element of the assessment for spectrum (left) and problem (right) elements across the 4 randomly selected demographically matched samples. Across all samples, the ratings were correlated with *r*>0.8 for all pairs, and the distributions of ratings for individual elements were highly similar and statistically indistinguishable (analysis of variance; *P*=.99), with an example from the element *Self-image* shown in Figure 1B. Similarly, the distributions of the resulting MHQ scores for each of these 4 samples were highly similar (Figure 1C, analysis of variance; *P*=.18). These results confirm that the MHQ, when offered anonymously and on the web, produces similar results across similar samples. Should responses have been randomly generated (eg, by bots) or if individuals had highly inconsistent interpretations of the life impact scale, this would not have been the case.

#### Internal Consistency

Among the related elements, the 2 questions relating to sleep quality and sleep sufficiency had a 0.63 correlation. Thus, those who had challenges with sleep quality were also likely to have fewer days of sufficient sleep. Similarly, the 2 questions relating to mood had a 0.64 correlation, indicating that those with a more significant impact of *Feelings of sadness, distress, or hopelessness* were also more likely to have a negative mood at the time of taking the assessment. Finally, the life impact rating of the MHQ element *Self-image* had a 0.77 correlation with the rating of the element *Self-worth and confidence*. In contrast, ratings of unrelated elements had lower correlations. For example, *Memory* and *Physical intimacy* had a correlation of 0.35, *Emotional control* and *Coordination* had a correlation of 0.36, and *Memory* and *Emotional control* had a correlation of 0.39. Therefore, related elements within the MHQ were more highly correlated than the unrelated elements examined.

#### Test-Retest Reliability

**Figure 2:**
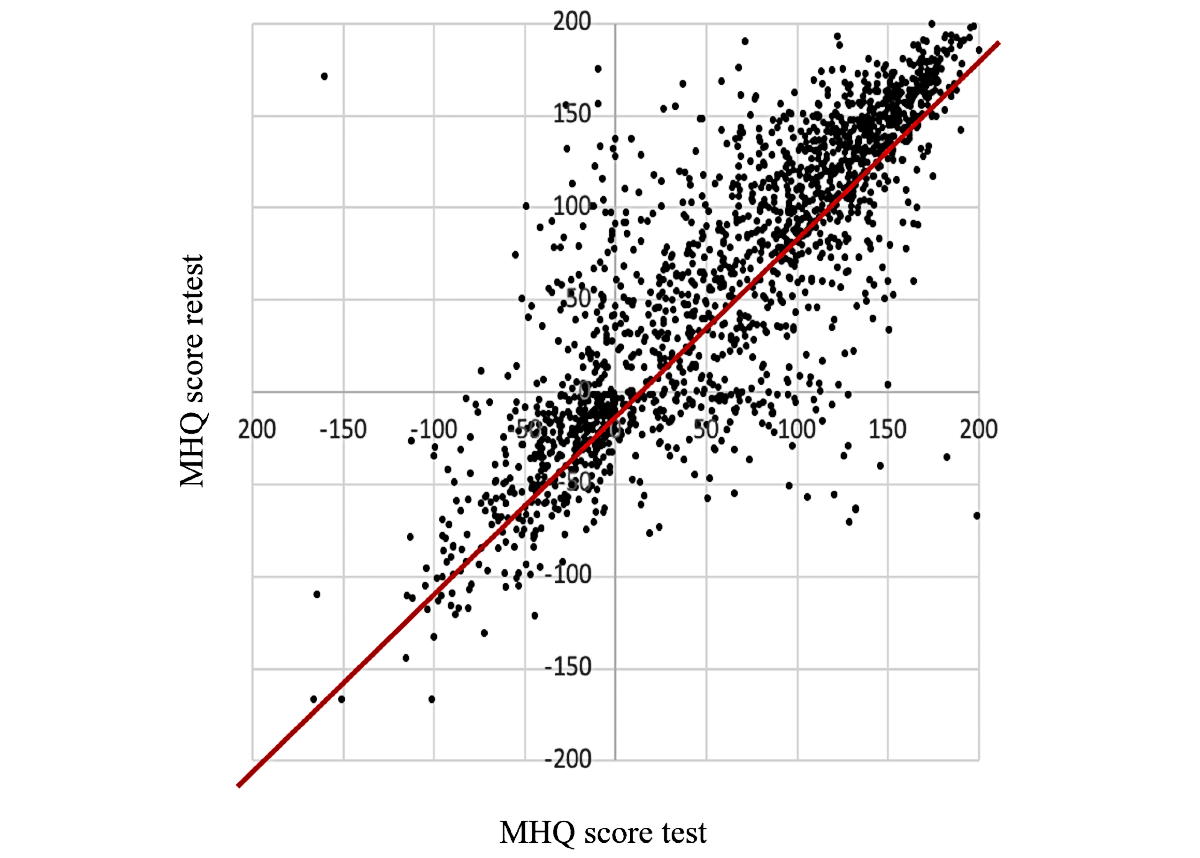
Test-retest reliability of the Mental Health Quotient (MHQ). MHQ score for the assessment retake (retest) versus MHQ score for the first take (test). The red line represents y=x. MHQ: Mental Health Quotient.

Among all those who could be identified as having taken the MHQ twice at least 3 days apart, MHQ scores were correlated with *r*=0.84 (*P*<.001). Figure 2 shows the MHQ scores for the test plotted against the MHQ scores for the retest, demonstrating that points fall around the line *y*=*x*. Furthermore, this correlation did not change significantly as the interval between attempts increased, although correlations were as high as *r*=0.88 for retest intervals of 8-120 days. The correlations (*r*) were as follows for MHQ scores and MHQ items ratings respectively: 3 to 7 days=0.7, 0.58; 8 to 30 days=0.88, 0.73; 31 to 60 days=0.88, 0.72; 61 to 120 days=0;.83, 0.7; 121 to 450 days=0.79, 0.68). Finally, the correlation between the ratings of individual elements on each attempt was *r*=0.70 (*P*<.001) and did not change as the interval between attempts increased. Thus, the MHQ had high test-retest reliability but also reflected changes that can occur in mental health and well-being over time.

### Relationship Between Life Impact Score and Frequency and Severity

**Figure 3:**
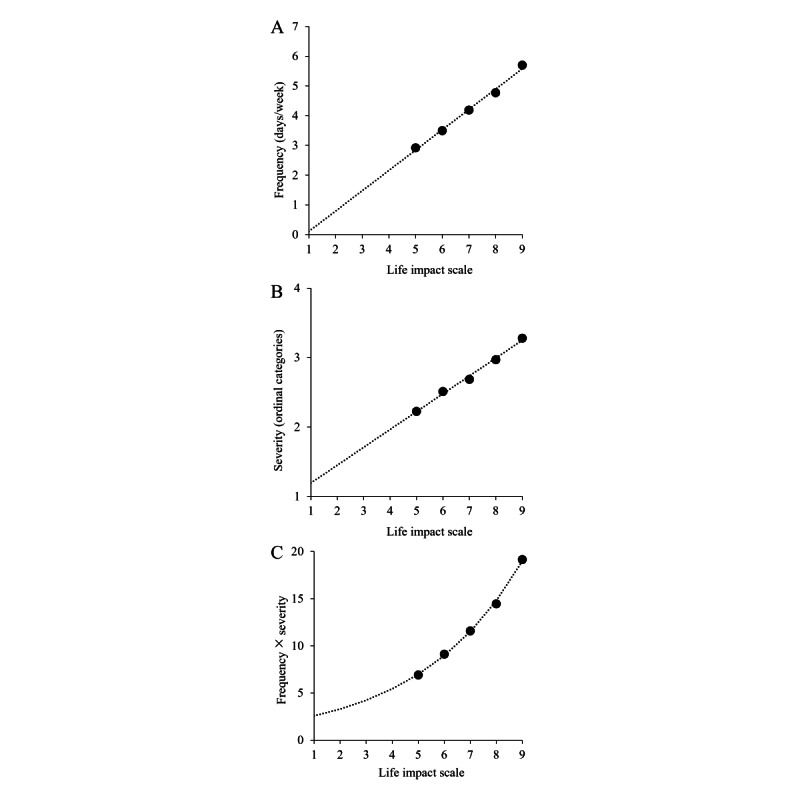
Example relationship between life impact and frequency and severity. (A) The selection on the Mental Health Quotient (MHQ) life impact scale for Feelings of sadness, distress, and hopelessness was linearly related to the frequency of these feelings. (B) The selection on the MHQ life impact scale was similarly linearly related to the severity of the symptom. (C) Frequency × severity was nonlinearly related to the MHQ life impact selection.

MHQ life impact ratings ≥5 (corresponding to the negative end of the scale) for the MHQ element *Feelings of sadness, distress, or hopelessness* (data subset of 4247/174,618, 2.43%) were correlated with symptom frequency measured as the number of days in the past week where they experienced the symptom, with *r*=0.5. At the aggregate level, the mean and SEM of frequency for each rating on the life impact scale were linearly related, with *R*^2^=0.99 (Figure 3A). Extrapolation of this function to life impact ratings <5 shows that those selecting 1 (the lowest end of the scale, indicating no impact) would have experienced that symptom at a frequency of <1 day in the previous week. Across all data, life impact was similarly positively correlated with the level of severity selected (where levels of severity were coded from 1 to 5) but less so than with frequency (*r*=0.32). However, the aggregate mean and SEM of severity for each life impact rating were also linearly related (Figure 3B; *R*^2^=0.99). Finally, in the aggregate, a composite measure of frequency × severity was nonlinearly related to the life impact rating, with *R*^2^=0.98 (Figure 3C). Therefore, we demonstrated a strong relationship between the rating on the life impact scale and both frequency and severity of symptoms.

### Relationship Between MHQ Score and Clinical Burden

**Figure 4:**
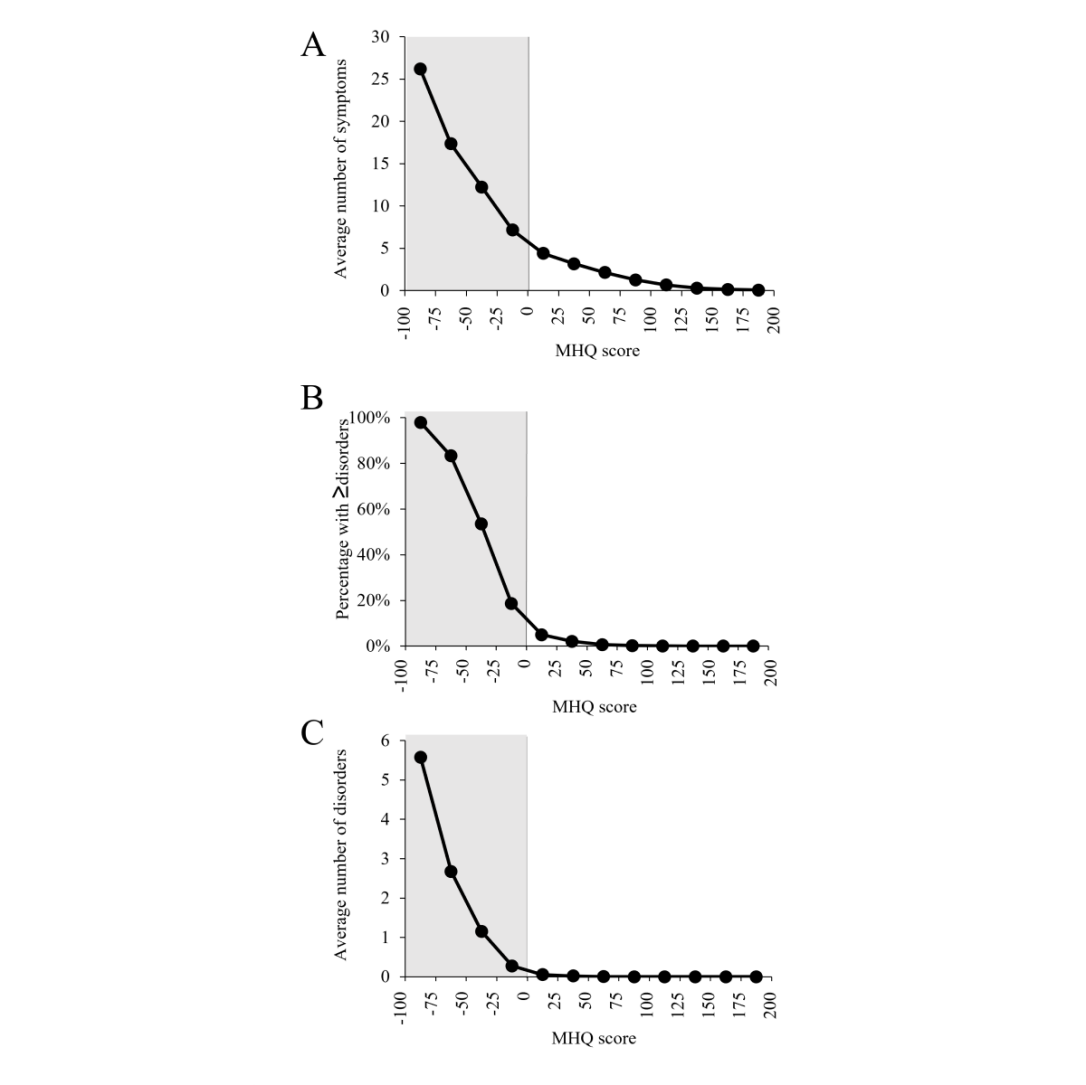
Relationship between Mental Health Quotient (MHQ) score and clinical symptoms and diagnosis. (A) The average number of symptoms (life impact ≥8 for problem elements and ≤1 for spectrum elements) decreased as the MHQ score increased. The grey area represents the negative side of the scale or the MHQ score categories of Distressed and Struggling (all panels). (B) Approximately 97.83% (3926/4013) of those with the lowest MHQ scores (−100 to −75) mapped to at least one of 10 major clinical disorders and decreased systematically. (C) The average number of disorders per person decreased with MHQ score.

First, as would be expected, the average number of clinical symptoms increased as the MHQ score decreased given the nonlinear weighting of symptom severity within the MHQ score (Figure 4A). Beyond this, the percentage of people with clinical symptom profiles that aligned with any of the 10 DSM-5–defined disorder criteria increased as the MHQ score decreased such that 89.08% (8986/10,087) of those in the Distressed category (MHQ score <−50) had symptom profiles that aligned with at least one of the 10 DSM-5–defined disorders, whereas 0.03% (21/70,367) in the categories of Succeeding and Thriving (MHQ score >100) had profiles that aligned with at least one disorder (Figure 4B). Similarly, the number of disorders per individual decreased systematically as MHQ scores increased, with the average number of disorders per person at 3.8 (SD 2.7) for those in the Distressed group and 0.0 (SD 0.02) for those in the Succeeding and Thriving groups (Figure 4C). Thus, the MHQ score is also reflective of the overall clinical burden of mental health.

### Loss of Function Criterion Validation

**Figure 5:**
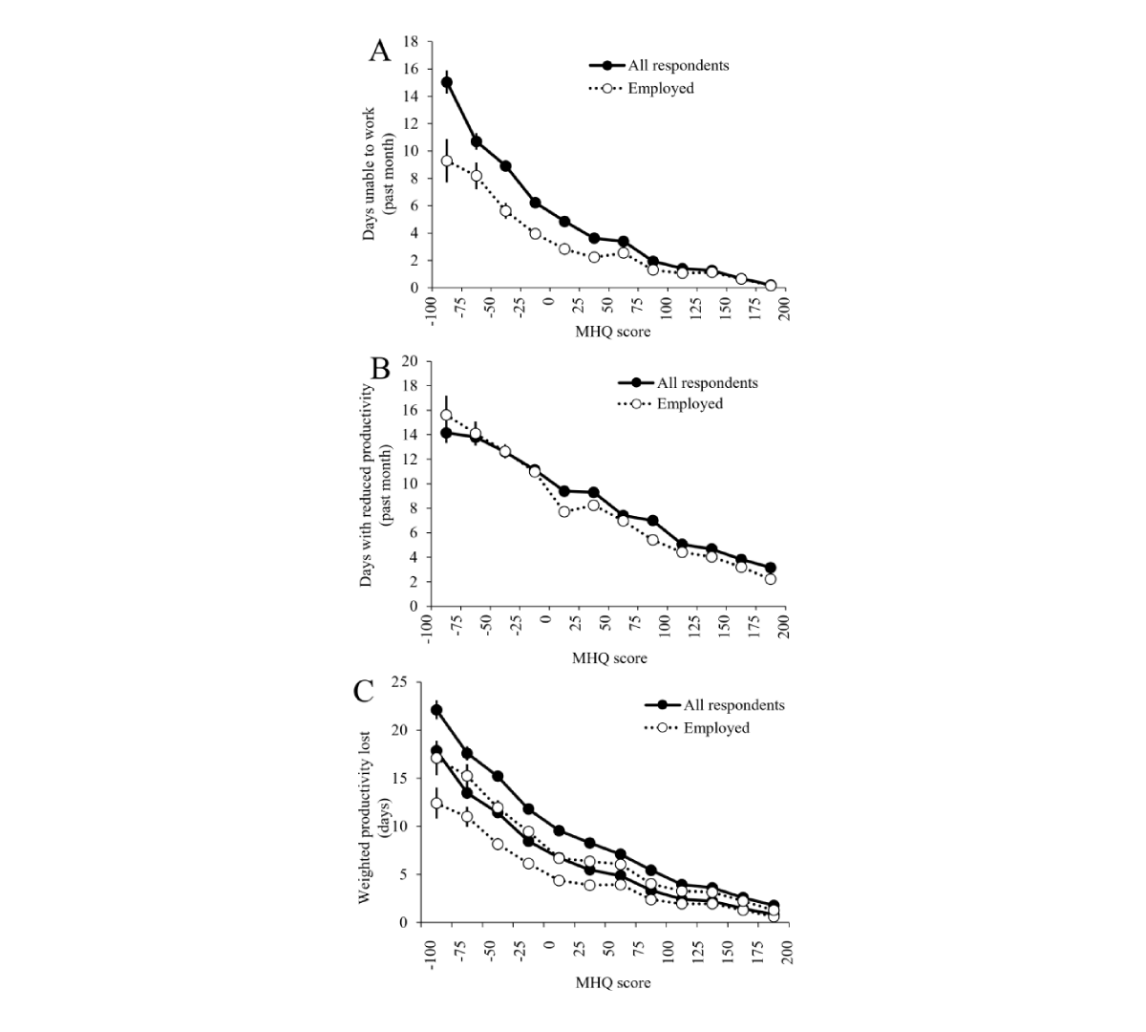
Relationship between Mental Health Quotient (MHQ) score and productivity. (A) The days unable to work in the past month decreased nonlinearly as the MHQ score increased (closed circles, exponential fit, *R*^2^=0.98). Employed people (open circles) with low MHQ scores missed fewer days of work or productive activity. (B) The days in the past month with reduced productivity (presenteeism) decreased linearly as the MHQ score increased (closed circles, exponential fit, *R*^2^=0.98). Employed people (open circles) with low MHQ scores had more days of presenteeism. (C) Total productivity loss for employed (dotted line) and all respondents together (solid line) as a function of the MHQ score (calculated as days missed + n * days with reduced productivity, where n is assumed to be a range between 0.2 [lower dotted or solid line] and 0.5 [upper dotted or solid line]).

As MHQ scores increased, the average number of days of work missed in the past month (Figure 5A) decreased systematically and was best fit by an exponential function, with *R*^2^=0.98. Those in the lowest MHQ score bin (−75 to −100) were unable to work or carry out their daily activities 15.0 (SD 11.3; SEM 0.9) days on average, whereas those who were employed (as opposed to studying, unable to work, unemployed, retired, or occupied with household work; 3306/174,618, 1.89%) were unable to work 9.3 (SD 10.0; SEM 1.6) days on average in the last month. In contrast, those who were in the highest MHQ bin (175-200) lost only an average of 0.2 (SD 1.6; SEM 0.1) days, whereas those who were employed lost an average of 0.2 (SD 0.54; SEM 0.07) days. Furthermore, as MHQ scores increased, the average number of days where people reported not being as productive as usual at work (presenteeism) or in their daily activities decreased linearly (Figure 5B; *R*^2^=0.98 for all respondents and employed respondents alone). Here, those in the lowest MHQ bin (−75 to −100) were not productive an average of 14.2 (SD 11.4; SEM 0.9) days, whereas those employed alone were not productive an average of 15.6 (SD 10.2; SEM 1.6) days. This decreased to an average of 3.2 (SD 9.5; SEM 0.8) and 2.2 (SD 8.5; SEM 1.1) days for all respondents and employed respondents, respectively, in the highest MHQ score bin (175-200). Figure 5C shows the total loss of life productivity as a function of the MHQ score considering both days of work missed and days that were less productive, assuming a range of 20% to 50% loss of productivity on less productive days. Altogether, those with the lowest MHQ scores had an overall reduction in life productivity of anywhere from 18 to 23 days per month on average. Although those with the highest MHQ scores did not often miss a day of work, even this group reported a few unproductive days per month. Thus, MHQ scores are a good representation of behavioral loss of function.

## Discussion

### Principal Findings

In this study, we have demonstrated that the MHQ taken anonymously on the web has excellent sample reliability, internal consistency, and test-retest reliability; that the life impact scale used in the MHQ reflects a combination of both severity and frequency of symptoms; and that the MHQ score relates systematically to clinical burden in the population as well as loss of function from the perspective of days of work missed and loss of productivity. Specifically, the results showed that (1) the MHQ scores were highly similar and statistically indistinguishable between multiple randomly selected, demographically matched samples of respondents; (2) MHQ scores were correlated between retakes with an *r*=0.84; (3) the life impact rating scale of the MHQ was systematically related to both symptom frequency and severity (*R*^2^=0.99); (4) ratings on related elements were more correlated than unrelated elements; and, finally, (5) MHQ scores decreased systematically with clinical burden and productivity (both *R*^2^=0.98). Thus, the MHQ provides a valid and reliable estimation of population mental health and well-being that, in turn, reflects the clinical burden of mental health and the productive capacity of a population. Therefore, it is also well-suited to measure changes in the status of mental health and well-being of a population. In addition, the test-retest reliability establishes the MHQ as a useful tool for individuals to track their mental health and well-being trajectory over time.

### MHQ and Clinical Burden

Surveillance of population mental health requires assessments that are reliable, valid, and accessible to the general population and that provide a comprehensive profile of mental health and well-being that has clinical and real-world relevance. Currently, many of the assessments used in epidemiological studies evaluate the prevalence of individual disorders rather than overall mental health and well-being. These tools typically consider mental illness as an all-or-nothing phenomenon, raising challenges relating to where the *border* between normal and disordered should lie [42-44] and leading to wide-ranging prevalence estimates that are dependent on the tool used and the thresholds considered as well as on geography and time period [18,45-51]. In addition, focusing only on individual disorders creates a siloed landscape of clinical burden that is at odds with the real-life heterogeneous and comorbid nature of symptomatic experiences and profiles [42,52-62]. Thus, generally, the aggregate burden of clinical-level mental health challenges beyond the domain of individual disorders is unknown in the general population. In this study, we have established the MHQ as a valid and reliable measure of mental health and well-being that can provide a view of overall mental distress and clinical burden. Rolled out at scale as it is currently being actioned as part of the Mental Health Million Project, the MHQ thus provides a solid foundation for the global surveillance of population mental health across different countries. This will help identify relevant risk factors to support the rollout of preventative strategies and the development of interventions or policies that could induce large-scale shifts in population well-being [8-10].

### MHQ and Productivity

Over the past few decades, there has been mounting evidence supporting the relationship between mental health and well-being and productivity [19-25] as well as the resultant economic loss to society as a consequence of days lost and unproductive days (eg, presenteeism) [28-31]. With the increased prevalence of mental distress as a result of the COVID-19 pandemic [16-18] and increased levels of burnout in the population [26,27], there is a need to better understand the relationship between mental health and well-being and productivity in the general population. The systematic relationship observed between the spectrum of MHQ scores from Distressed to Thriving and productivity loss along with the general reliability and validity of the MHQ support its use as an assessment of the productive capacity of a population independent of any disorder classification. It also positions the MHQ as an important tool for companies to assess the mental health and well-being of their workforce, providing relevant metrics that can help them address challenges such as employee burnout and work-home imbalance [26,27], as well as for university student bodies, where young adults are disproportionally affected by mental health challenges [63-65]. This will allow and encourage organizations and institutions to be more strategic in their management of mental health and well-being.

### Limitations and Future Directions

It is important that we acknowledge some limitations of these data and study. First, the validation of the life impact rating against symptom frequency and severity was performed for a single MHQ element (*Feelings of sadness, distress, or hopelessness*). However, it is possible that the correspondence between frequency or severity and life impact rating may differ from element to element. Furthermore, these results were used to select an appropriate threshold value for clinical significance [42] to determine clinical burden, indicating that a threshold of 8 was equivalent, on average, to experiencing the symptom 5 days per week. However, it could be the case that other threshold values may have been more appropriate for other elements.

Second, the mapping of MHQ elements to DSM-5 diagnostic criteria was constrained by the presence of broad or imperfect matches for certain symptoms pertaining to OCD and bipolar disorder that could have affected the accuracy of the mapping [42]. For example, for bipolar disorder, symptoms denoting extreme versions of positive assets (eg, *grandiosity and decreased need for sleep*) were not fully articulated within the MHQ, whereas, for OCD, the MHQ elements were broader (eg, obsessive thoughts were incorporated within a general element reflecting strange, unwanted, and obsessive thoughts). Furthermore, a specific criterion of symptom timing was not included as this is not included in the MHQ, which assesses an individual’s current perception.

Third, in the future, it will be important to compare the MHQ outcomes with more commonly used assessments (eg, mapping against the 9-item Patient Health Questionnaire [66] and the 7-item Generalized Anxiety Disorder scale [67]) to determine the alignment between DSM-5–mapped MHQ symptom profiles for depression and anxiety and the scores from these 2 questionnaires, respectively. Triangulating data arising from the Mental Health Million Project against other external data metrics that provide insight into clinical burden (eg, quality-adjusted life years or disability-adjusted life years) will also be important.

Altogether, the MHQ supports a valid and reliable monitoring of population mental health and well-being. As the MHQ continues to underpin large-scale initiatives such as the Mental Health Million Project, it will provide deeper insights into social determinants and the societal impact of changes in mental health and well-being. These insights can, in turn, enable preventative strategies for better management of global mental health and well-being.

## Abbreviations

DSM-5: Diagnostic and Statistical Manual of Mental Disorders, Fifth Edition
MHQ: Mental Health Quotient
OCD: obsessive-compulsive disorder
SEM: standard error of measurement
